# Inherited Thrombocytopenia Related Genes: GPS2 Mediates the Interplay Between ANKRD26 and ETV6

**DOI:** 10.3390/cells14010023

**Published:** 2024-12-30

**Authors:** Valeria Capaci, Melania Eva Zanchetta, Giorgia Fontana, Daniele Ammeti, Roberta Bottega, Michela Faleschini, Anna Savoia

**Affiliations:** 1 Institute for Maternal and Child Health, IRCCS Burlo Garofolo, 34137 Trieste, Italy; melaniaeva.zanchetta@burlo.trieste.it (M.E.Z.); giorgia.fontana@burlo.trieste.it (G.F.); daniele.ammeti@burlo.trieste.it (D.A.); roberta.bottega@burlo.trieste.it (R.B.); michela.faleschini@burlo.trieste.it (M.F.); 2 Department of Neurosciences, Biomedicine and Movement Sciences, University of Verona, 37134 Verona, Italy

**Keywords:** ANKRD26, ETV6, GPS2, hematological neoplasms, thrombocytopenia

## Abstract

Mutations in the genes *ANKRD26*, *RUNX1*, and *ETV6* cause three clinically overlapping thrombocytopenias characterized by a predisposition to hematological neoplasms. The *ANKRD26* gene, which encodes a protein involved in protein-protein interactions, is downregulated by RUNX1 during megakaryopoiesis. Mutations in 5′UTR of ANKRD26, leading to ANKRD26-RT, disrupt this regulation, resulting in the persistent expression of ANKRD26, which leads to impaired platelet biogenesis and an increased risk of leukemia. Although ANKRD26 and ETV6 exhibit inverse expression during megakaryopoiesis, ETV6 does not regulate the ANKRD26 expression. Hypothesizing an interplay between ETV6 and ANKRD26 through in vitro studies, we explored the interactions between the two proteins. In this study, we found that ANKRD26 interacts with ETV6 and retains it in the cytoplasm, phenocopying ETV6-RT-related mutants. We found that GPS2, a component of the NCoR complex, binds both ANKRD26 and ETV6, mediating this interaction. Furthermore, ANKRD26 overexpression deregulates ETV6 transcriptional repression, supporting a common pathogenic mechanism underlying ANKRD26-RT, FPD/AML, and ETV6-RT. Our results unveil a novel ANKRD26-ETV6-GPS2 axis, providing new insights to investigate the molecular mechanism underlying thrombocytopenias with a predisposition to myeloid neoplasms that need to be further characterized.

## 1. Introduction

Germline mutations of the *ANKRD26*, *RUNX1*, and *ETV6* genes are associated with three autosomal dominant forms of thrombocytopenias: *ANKRD26*-Related Thrombocytopenia (*ANKRD26*-RT), Familial Platelet Disorder with propensity to Acute Myelogenous Leukemia (FPD/AML) and *ETV6*-Related Thrombocytopenia (*ETV6*-RT). They are characterized by moderate thrombocytopenia with platelets of normal size, mild or absent bleeding tendency, and an increased risk of developing hematological neoplasms [1], including myelodysplastic syndrome, acute myeloid leukemia, and acute lymphoblastic leukemia.

The *ANKRD26* gene encodes a protein of poorly characterized functions. It contains five N-terminal ankyrin repeats and a helical region structurally similar to α-spectrin, suggesting that it might play a role in complexes as an adaptor for protein-protein interactions, as supported by the identification of a plethora of interactors, including TRIO, DIPA, GPS2, FBF1, PIDD1 [2,3,4,5,6]. During megakaryopoiesis, the *ANKRD26* gene is physiologically downregulated by RUNX1, a transcription factor master regulator of hematopoietic cell development, and indeed, its levels progressively decline with almost undetectable expression in mature megakaryocytes (MKs), and in platelets [7].

Consistently, the mutations identified in ANKRD26-RT affected individuals are all clustered in a short specific stretch in the 5′-untranslated region (5′-UTR) containing the binding site for RUNX1, preventing its downregulation required for a proper megakaryocyte differentiation [8]. Interestingly, an increased expression of ANKRD26 was also reported in a family whose thrombocytopenia was caused by a complex structural variant generating a WAC-ANKRD26 fusion transcript [9], suggesting that the loss of ANKRD26 downregulation is a common mechanism in Its, including ANKRD26-RT and FPD/AML. Persistent ANKRD26 expression in MKs sustains the MAPK/ERK1/2 signaling pathway hyperactivation, determining the impairment of platelet biogenesis [7] and increased risk of hematological malignancies observed in these patients [10].

The *ETV6* gene encodes a transcription factor, a member of the ETS family playing a role in hematopoiesis, mainly as a transcriptional repressor in complexes with different nuclear corepressors and epigenetic modifiers, such as SIN3A, NCOR, and HDAC3 [11,12,13,14]. The ETV6 protein is characterized by two highly conserved domains: the Pointed (PNT) domain, essential for homo- and hetero-oligomerization, and the ETS domain, responsible for DNA binding. At least 15 different heterozygous germline mutations of *ETV6*, mainly missense amino acid substitutions affecting the ETS domain, have been associated with thrombocytopenia [15,16]. The mutant forms of the transcription factor are unable to enter the nucleus and exert dominant-negative activity over the wild-type form [14,16,17]. Of note, the abnormal cytoplasmic localization of ETV6 mutants is associated with the mislocalization of the HDAC3/NCOR2 repressor complex and induction of interferon response genes driving aberrant proplatelet formation in MKs [14].

To date, the pathogenetic mechanisms responsible for thrombocytopenia and the neoplastic evolution in ANKRD26-RT, FPD/AML, and ETV6-RT are still unknown. Since these diseases are phenotypically identical, we hypothesized an interplay between ETV6 and ANKRD26 and found that ANKRD26 interacts with ETV6 through GPS2, preventing the transcription factor from entering the nucleus to exert its functions and suggesting a common mechanism in the pathogenesis of these three tumor-predisposing inherited thrombocytopenias.

## 2. Material and Methods

### 2.1. Cell Culture and Transfection

HEK293T and HeLa cells were grown in a DMEM medium, while K562 and DAMI cells were cultured in RPMI. The base media were supplemented with 10% fetal bovine serum (FBS), penicillin, and streptomycin (100 IU/mL each). All cell lines were cultured in a 37 °C incubator with 5% CO_2_.

Transfections were carried out when the cultures reached 50% to 80% confluence. For DNA transfections, the required amount of DNA was used in combination with PEI (Polyethylenimine branched, Sigma Aldrich, Merck KGaA, Darmstadt, Germany, 408727), Lipofectamine 3000 (Invitrogen™, Life Technologies, #L3000001), or Lipofectamine LTX (Invitrogen™, Life Technologies, Thermo Fisher, Waltham, MA, USA, #15338500), following the manufacturer’s protocols. SiRNAs were transfected at a final 100 nM concentration using Lipofectamine RNAiMax (Invitrogen™, #13778075) according to the manufacturer’s instructions, and transfected cells were incubated for 48 h prior to analysis. Eurofins Control siRNA sequence was used as the negative control. Details of the siRNA sequences are provided in Table 1.

### 2.2. RNA Extraction and Quantitative Real-Time PCR

Total RNA was extracted using the High Pure RNA Isolation Kit (Roche, Basel/Kaiseraugst, Switzerland, #11828665001), followed by the analysis of concentration, quality, and purity assessed with the NanoDrop ND-1000 Spectrophotometer (NanoDrop Technologies Inc. Wilmington, DE, USA). For qPCR analysis, 1 μg of total RNA was retrotranscribed using the iScript™ Advanced cDNA Synthesis Kit (Bio-Rad, Hercules, CA, USA, #1725037). Quantitative gene expression analysis was performed with iTaq™ Universal SYBR^®^ Green Supermix (Bio-Rad, #1725120) on the Applied Biosystems 7500 FAST DX system (Thermo Fisher). Data were processed with the 7500 Fast Real-Time PCR System Software v2.3 (Thermo Fisher).

Experiments were conducted at least three times, with each sample analyzed in technical duplicates. Gene expression quantification was calculated based on the 2^−ΔΔCt^ method, normalizing the expression to housekeeping genes. Primer sequences are listed in Table 2.

### 2.3. Western Blot and Coimmunoprecipitation (Co-IP) Experiments

Cells were lysed in lysis buffer (300 mM NaCl, 50 mM Tris-HCl, pH 7.5, 1 mM EDTA, 1% NP-40), supplemented with protease and phosphatase inhibitors (1 mM PMSF, 5 mM NaF, and 1 mM Na_3_VO_4_) and then protein concentrations were measured using the Bio-Rad Protein Assay Reagent (Bio-Rad, #500-0006). Proteins were resolved by SDS-PAGE, transferred onto nitrocellulose membranes (Bio-Rad, #1704158), and analyzed by standard Western blotting procedures.

For Co-IP experiments, cells were lysed using a buffer lysis buffer containing 150 mM NaCl, 50 mM Tris-HCl (pH 8), 1 mM EDTA, and 1% NP-40, supplemented with protease inhibitors, and then the lysates clarified by centrifugation at 13,000× *g* for 5 min at 4 °C. Lysate was incubated for 3 h at 4 °C with the specific or unrelated antibody. Subsequently, the samples were incubated for 1 h with protein A/G PLUS Agarose (Santa Cruz Biotechnologies, Santa Cruz, CA, USA, sc-2003). Immunoprecipitates were washed three times in Co-IP lysis buffer, and then resuspended in Laemmli sample buffer, and analyzed by Western blotting.

Antibodies used are detailed in Table 3. Images were captured using the ChemiDoc MP Imaging System (Bio-Rad), and band intensities were quantified using FIJI software v2.14.0/1.54f (NIH Image, Bethesda, MD, USA) [17].

### 2.4. Dual Luciferase Assays

HEK293T cells were plated in 24-well plates and transfected with 100 nM GPS2 specific siRNAs or negative control. After 24 h, cells were co-transfected with 300 ng of pGL3-MMP3 reporter vectors and 100 ng of pRL-CMV, and the medium was replaced 6 h post-transfection. The luciferase activity was measured 48 h later using the Dual-Luciferase^®^ Reporter Assay System (Promega, Madison, WI, USA, #E1910) kit by the Glomax Discover instrument (Promega).

For each sample, the Relative Luciferase Units (RLU) were calculated by normalizing the Firefly to Renilla luciferase activity.

### 2.5. Proximity Ligation Assay

HeLa cells were plated on coverslips and fixed after 48 h with 4% paraformaldehyde for 15 min. Cell permeabilization was performed with 0.1% Triton X-100 for 5 min. Then, the Duolink In Situ Red Starter Kit Mouse/Rabbit kit (Sigma-Aldrich, #DUO92101) was used to perform PLA assay following the manufacturer’s protocol and analyzed with the Zeiss Axioplan 2 epifluorescence microscope (Oberkochen, Germany). Representative images were acquired for each biological group. Localization analysis of the Duolink signal, along with nuclei stained with DAPI, was performed using the Pearson’s r correlation coefficient calculated via the JACoP plug-in in the FIJI software v2.14.0/1.54f (NIH Image) [17,18].

### 2.6. Immunofluorescence

Cells were fixed in 4% paraformaldehyde for 15 min, washed in PBS, permeabilized with Triton 0.1% for 10 min, and blocked in FBS 3% in PBS for 30 min. Antigen recognition was performed by incubating the primary antibody for 2 h at 37 °C and with the secondary antibody for 45 min at 37 °C. Nuclei were counterstained with DAPI (Life Technologies, Thermo Fisher, #10236276001). The antibodies used are listed in Table 3.

### 2.7. Plasmids

The pGL3-MMP3, pCDNA3-Myc-ETV6, pCDNA3-Myc-ETV6, pCDNA3-Myc-ETV6Q347P and pCDNA3-Myc-ETV6W380R were previously described [16].

The p3XFLAG-CMV-ANKRD26 vector was kindly provided by Ilaria Cannobia and obtained as previously described [19].

### 2.8. Statistical Analyses and Reproducibility

All experiments were conducted with a minimum of three independent replicates. Data in all graphs are presented as the mean of individual data points ± SEM. Statistical analyses were performed using GraphPad Prism 8 (GraphPad Software LLC, Boston, MA, USA) with *p*-values calculated via a two-tailed Student’s unpaired parametric *t*-test. The reported micrographs and blots are representative of three independent experiments.

## 3. Results and Discussion

### 3.1. Unraveling the Interplay Between ANKRD26 and ETV6

The similar clinical picture of the ANKRD26-RT, FPD/AML, and ETV6-RT inherited thrombocytopenia [7], elicits to hypothesize a crosstalk among the proteins encoded by the genes responsible for the three diseases. Since the transcriptional regulation of ANKRD26 by RUNX1 has already been established [7], we wondered whether there might be an interplay between ETV6 and ANKRD26 that had never been investigated before.

**Figure 1:**
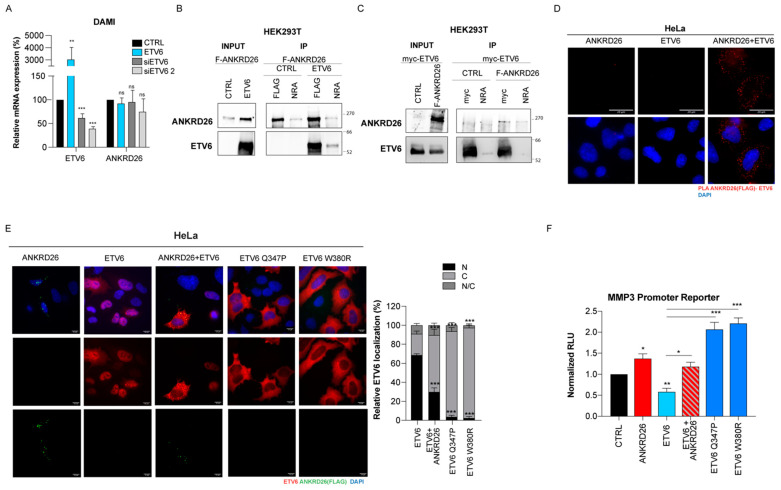
Unraveling the interplay between ANKRD26 and ETV6: (**A**). Expression levels of the ETV6 and ANKRD26 transcripts determined by qRT-PCR in DAMI cells upon the overexpression of ETV6 or silencing of ETV6 using two specific siRNAs normalized to the ACTB (beta-Actin) gene expression. (**B**,**C**). Western blot analysis of co-immunoprecipitation of ETV6 and ANKRD26 in HEK293T cells transfected with myc-ETV6 and FLAG-ANKRD26 alone or in combination for 48 h. NRA: not related antibody. (**D**). Representative images of proximity ligation assay (PLA) experiments using anti-ETV6 and anti-FLAG antibodies in HeLa cells after the overexpression of myc-ETV6 and FLAG-ANKRD26 alone or in combination for 48 h. (**E**). Representative images of the immunofluorescence analysis of ETV6 (anti-ETV6, red) and ANKRD26 (anti-FLAG, green) in HeLa cells overexpressing myc-ETV6 and FLAG-ANKRD26. Two mutant forms (Q347P and W380R) of myc-ETV6 were also transfected as a control for cytoplasmic localization. Nuclei were marked with DAPI staining (blue). Scale bar, 20 µm. Right: graph showing the percentage of cells with Nuclear (N), cytoplasmic (C), or both (N/C) ETV6 localization upon different conditions. (**F**). Luciferase assay performed on HEK293T cells after the overexpression of ANKRD26 and/or ETV6, wt, or mutated as indicated. Firefly luciferase cloned downstream MMP3 promoter was used as reporter, and Renilla luciferase under the control of CMV promoter as normalizer. The graph represents the mean ± SEM of three independent experiments. The *p*-value (* *p* < 0.05, ** *p* < 0.01, *** *p* < 0.001) was calculated by a two-tailed unpaired Student’s *t*-test.

Interestingly, our results show that the *ETV6* and *ANKRD26* genes are inversely expressed in K562, MEG-01, and DAMI cells, three cellular models representing different stages of megakaryopoiesis (hematopoietic stem cells potential, immature and mature MKs, respectively) (Supplementary Figure S1A). Based on these observations and considering that it is a transcriptional repressor, we hypothesized that ETV6, like RUNX1, might be involved in the regulation of *ANKRD26* expression. However, either the knockdown or ectopically overexpression of ETV6 did not affect mRNA ANKRD26 expression both in DAMI (Figure 1A) and HEK-293T cells (Supplementary Figure S1B).

Having ruled out the transcriptional regulation of *ANKRD26* by ETV6 and considering that ANKRD26 functions as a scaffold protein involved in the regulation and assembly of multimeric complexes, we hypothesized that crosstalk between ANKRD26 and ETV6 might occur through protein-protein interactions.

To address this, we performed a coimmunoprecipitation (Co-IP) assay in HEK293T cells after the overexpression of myc-ETV6 and FLAG-ANKRD26 proteins; the immunoblot analysis confirmed that ANKRD26 and ETV6 coimmunoprecipitated when the assay was performed with both myc-tag (ETV6) and FLAG (ANKRD26) antibodies (Figure 1B,C) suggesting that the two proteins are subunits of the same multimeric complex.

To better characterize this interaction, we performed proximity ligation assays (PLA) in HeLa cells, overexpressing the two tagged proteins alone or in combination. The presence of multiple dots in the cytoplasm of HeLa cells revealed that ANKRD26 and ETV6 colocalize in this subcellular compartment (Figure 1D).

Since ANKRD26 localizes within the cytoplasm [6,20] and ETV6 shuttles between the cell nucleus and cytoplasm to exert its functions, we wondered whether ANKRD26 overexpression might affect the ETV6 localization balance. We thus performed an immunofluorescence assay in transiently transfected HeLa cells, confirming that ANKRD26 accumulates in dots within the cytoplasm, while the ETV6 is mainly localized in the nuclear compartment (69% of cells). Interestingly, the coexpression of ANKRD26 and ETV6 induced a mislocalization of the transcription factor, which is retained in the cytoplasm in this condition (60% of cells), analogous to two mutant forms of ETV6 (Q347P and W380R) whose cytoplasmic retention has been previously demonstrated (Figure 1E). These data suggested that the mislocalization induced by the overexpression of ANKRD26 could affect ETV6 transcriptional activity, as has been shown for ETV6 mutations. To verify this hypothesis, we took advantage of a previously described reporter assay to evaluate the repressive activity of ETV6 [21]. As expected, the ETV6 overexpression in HEK293T cells induced a significant repression of the luciferase activity (50%) under the control of the MMP3 promoter, which is not observed after the overexpression of the Q347P and W380R ETV6 mutants (Figure 1F). Interestingly, the overexpression of ANKRD26 slightly induces the MMP3 promoter activity (red bar). More importantly, the ETV6 inhibitory activity on the MMP3 promoter is abrogated when *ANKRD26* is also co-expressed (Figure 1F).

Consistent results in the megakaryopoietic DAMI cellular model, the ETV6 overexpression repressed MMP3 expression, while W380R ETV6 mutant and ANKRD26 overexpression slightly induced it (Supplementary Figure S2).

Taken together, these results suggest that ANKRD26 overexpression sequesters the transcription factor in the cytoplasm, leading to an impairment of ETV6 repression activity, suggesting a similar molecular mechanism in tumor-predisposing inherited thrombocytopenias.

### 3.2. GPS2 Mediates ANKRD26/ETV6 Interaction

**Figure 2:**
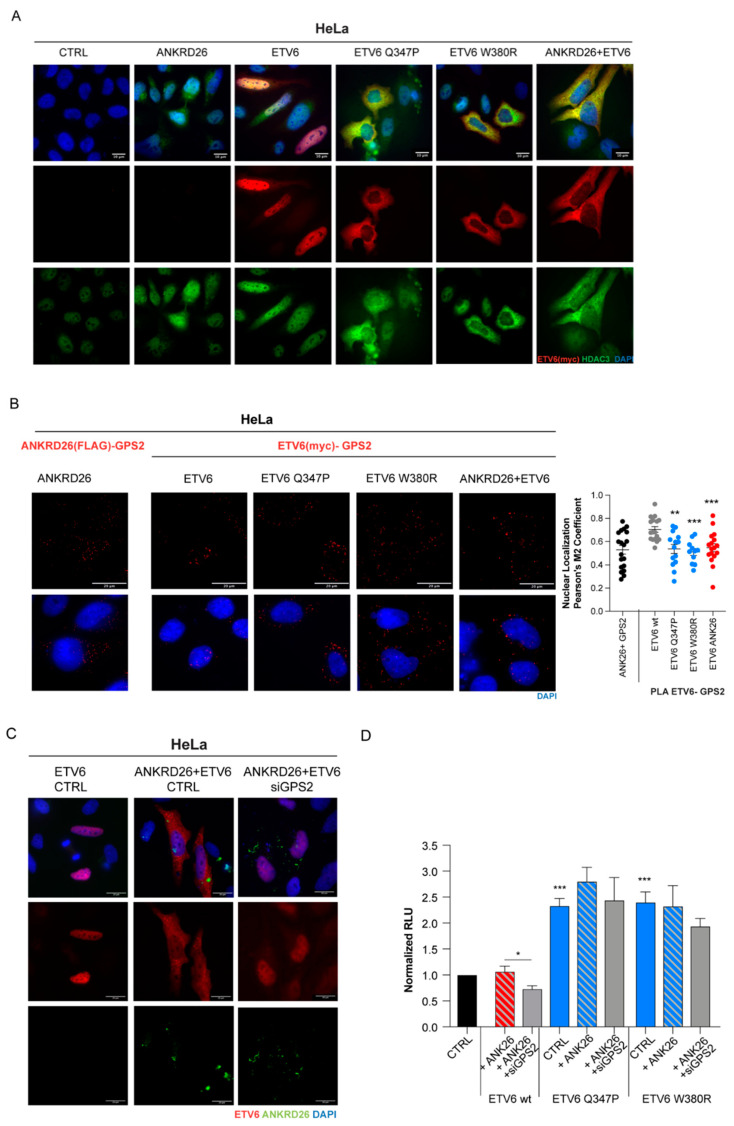
GPS2 mediates the ANKRD26 and ETV6 interaction: (**A**). HeLa cells were transfected with myc-ETV6 (either wt or mutant Q347P and W380R forms) and FLAG-ANKRD26 expression constructs alone or in combination. Representative images of the immunofluorescence analysis of ETV6 and HDAC3 detected using anti-myc (red) and anti-HDAC3 (green) antibodies, respectively. Nuclei were marked with DAPI staining (blue). Scale bar, 20 µm. (**B**). Representative images of PLA between GPS2 and ANKRD26 (using GPS2 and FLAG- tag primary antibodies), or between GPS2 and ETV6 (using GPS2 and myc-tag primary antibodies) in HeLa cells transfected as in (**A**) Right: graph showing the Pearson’s correlation coefficient of PLA signal localization in the nucleus (n = 20 cells for each condition). (**C**). Representative images of the immunofluorescence analysis of ETV6 in HeLa cells overexpressing myc-ETV6 alone, as well as in combination with FLAG-ANKRD26 combined with the silencing of endogenous GPS2. Myc-ETV6 was detected using antibodies against ETV6 (red) and ANKRD26 with anti-FLAG (green) antibodies. Nuclei were marked with DAPI staining (blue). (**D**). Luciferase assay performed on HEK293T cells upon the overexpression of ANKRD26 and/or ETV6, either wt or mutant as indicated, combined with knockdown of endogenous GPS2 with a specific siRNA. Firefly luciferase cloned downstream MMP3 promoter was used as reporter, and Renilla luciferase under the control of CMV promoter as normalizer. The graph represents the mean ± SEM of three independent experiments. The *p*-value (* *p* < 0.05, ** *p* < 0.01, *** *p* < 0.001) was calculated by a two-tailed unpaired Student’s *t*-test.

A recent report showed that abnormal cytoplasmic localization of mutant ETV6 forms is associated with the mislocalization of the HDAC3/NCOR2 repressor complex and induction of interferon response genes, driving aberrant proplatelet formation in MKs [14]. Given the ability of ANKRD26 to sequester ETV6 in the cytoplasm, we wondered whether ANKRD26 can affect the HDAC3 localization. Therefore, we performed immunofluorescence assays and found, as expected, that HDAC3 was prevalently localized in the nuclei of control cells or cells overexpressing wild-type (wt) ETV6 while in the cells overexpressing the two mutant forms Q347P and W380R, it is mainly present in the cytoplasmatic compartment. Interestingly, the concomitant expression of ANKRD26 and ETV6 not only caused the mislocalization of ETV6 but also of HDAC3 (Figure 2A).

Of note, the NCoR/HDAC3 complex represents one of the major complexes involved in gene silencing and maintaining the heterochromatin regions of DNA [22,23,24,25] and consists of six core subunits: HDAC3, NCOR, NCOR2 (also known as SMRT), GPS2, TBL1, and TBLR1. Among them, GPS2, NCOR, and TBL1 promote the assembly and stabilization of the whole complex [25].

Interestingly, GPS2 is one known interactor of ANKRD26 [6], and since a direct interaction between ANKRD26 and ETV6 has not been previously described, we hypothesized that the GPS2 protein could mediate their binding.

To unveil this, we performed PLA experiments in HeLa cells overexpressing ANKRD26 confirming that GPS2 forms complexes with ANKRD26 as previously reported (Figure 2B). Moreover, the interaction between GPS2 and ETV6 was observed with both wt-ETV6 and its mutant forms mainly in the nucleus or in the cytoplasmatic compartment, respectively (Figure 2B). Interestingly, when ANKRD26 and ETV6 were co-overexpressed, the ETV6/GPS2 complex was retained into the cytoplasm, as observed after the overexpression of the mutant forms of ETV6 (Figure 2B).

To confirm the role of GPS2 in ANKRD26/ETV6 interaction, we co-transfected ETV6 and ANKRD26 alone or combined with GPS2 siRNA and immunoprecipitated ETV6. Immunoblotting showed that the amount of ANKRD26 co-immunoprecipitated was significantly reduced when GPS2 is silenced, confirming that GPS2 is a mediator of their interaction (Supplementary Figure S3).

Consequently, our results indicated that the knockdown of GPS2 partially restored the nuclear localization of ETV6 (Figure 2C) despite ANKRD26 overexpression, supporting our hypothesis that the three proteins are subunits of the same multimeric complex.

Of note, by immunofluorescence, we showed that the silencing of GPS2 also affects ANKRD26 localization, as the staining is localized in the peri membranous region instead of dots within the cytoplasm. Altogether these data suggest that GPS2 is essential to finely tune the ANKRD26 and ETV6 localization and indeed to promote ETV6/ANKRD26 complex formation, whose function impact still need to be clarified.

Next, we investigated the functional effect of GPS2 on the ANKRD26-dependent modulation of ETV6 transcriptional activity. Importantly, we found that GPS2 depletion abrogates the de-repression promoted by ANKRD26 on the MMP3 promoter, restoring the correct transcriptional activity of ETV6 (Figure 2D).

Consistent with the di per se cytoplasmic localization of ETV6 mutated proteins, their transcriptional activity is not modulated by ANKRD26 overexpression neither alone nor in combination with the silencing of GPS2 (Figure 2D).

Overall, these data support a role for ANKRD26 in controlling the repressive function of ETV6 through the GPS2-mediated retention of the transcription factor in the cytoplasm.

Taken together, our data suggest that impaired ETV6 transcriptional activity due to its cytoplasmic retention is the common pathogenetic mechanism that may explain how tumor-predisposing inherited thrombocytopenias phenocopy each other. Indeed, whereas in ETV6-RT, the aberrant ETV6 localization is directly caused by mutations, our results suggest that in *ANKRD26*-RT and probably in FDP/AML, it is determined by an abnormal overexpression of ANKRD26 which, via GPS2, causes the mislocalization of the wt protein; however further studies in patients-derived samples are needed to broaden evidence provided in this work.

Since ANKRD26 must be downregulated during megakaryopoiesis for proper platelet biogenesis, we speculate that when this repression is lacking (due to mutations in the ANKRD26 or RUNX1 genes), the transcriptional program of ETV6 may be altered. Moreover, we hypothesize that in this condition, the HDAC3/NCOR2 repressor complex could also be retained in the cytoplasm, possibly leading to an aberrant epigenetic profile similar to that already reported for ETV6 mutants [14].

Although there is currently no treatment for ETV6-RT, ANKRD26-RT, and FDP/AML, the molecular data accumulated over time suggests different levels of regulation that could potentially be targeted. For ANKRD26, the ideal strategies would involve a direct modulation of its expression or, downstream, the inhibition of the MAPK pathway hyperactivation. In the case of mutant forms of ETV6, effective strategies might aim to restore proper ETV6 nuclear localization. However, implementing these approaches at the molecular level remains challenging.

This work emphasizes the hypothesis that the pathogenetic mechanism underlying not only ETV6-RT but also ANKRD26-RT and possibly FDP/AML may involve a shared impaired epigenetic profile. This is particularly intriguing because GPS2/NCoR-dependent epigenetic modifications are druggable, suggesting new opportunities for precision medicine in the treatment of ‘myeloid neoplasms with germline predisposition and preexisting platelet disorders’, as the three diseases have been classified [26].

Furthermore, potential strategies might include uncoupling ANKRD26 from ETV6 or interfering with their downstream signaling pathways, such as epigenetic rewiring or the MAPK pathways.

## Figures and Tables

**Table 1:** List of siRNAs used in the present study.

Target	Sequence	Reference
CTRL	5′-AGGUAGUGUAAUCGCCUUG-3′	
ETV6_1	5′-AATTTACTGGAGCAGGGATGA-3′	
ETV6_2	5′-AAGAGGACTTTCGCTATCGAT-3′	
ANKRD26	5′-GAAAGAAGTTGAAGTGAAA-3′	Bluteau, 2015 [6]
GPS2	5′-GUGACCAUCAGAUUAUAUCTT-3′	

**Table 2:** List of oligonucleotides used in the present study.

qPCR Primers		
Target	Primer Sequence (5′-3′)	Sense
Actin	CAACACAGTGCTGTCTGGC	FW
Actin	GGAGCAATGATCTTGATCTTC	RV
ANKRD26	GACCGAGATCTCGGCAAG	FW
ANKRD26	GGCATTGTACAGCCTTCATC	RV
ETV6	TCTTAAATGACCGCGTCTGGC	FW
ETV6	GAGGAAGCGTAACTCGGCAC	RV
GPS2	AAACGGAGGCGAAAGGAACA	FW
GPS2	AGCACTTGGGGTCCAAACAT	RV
MMP3	TGAGGACACCAGCATGAACC	FW
MMP3	ACTTCGGGATGCCAGGAAAG	RV
RUNX1	TGCGGCGCACAGCCATGA	FW
RUNX1	AGATGATCAGACCAAGCCCG	RV

**Table 3:** List of antibodies used in the present study.

Target Protein Name	Producer	ID Number	WB Dilution	IF Dilution
GAPDH	Santa Cruz Biotechnology	sc-47724, RRID:AB_627678	1:3000	
HSP90 alpha/beta (F-8)	Santa Cruz Biotechnology	sc-13119; RRID: AB_675659	1:3000	
ANKRD26	Genetex	GTX128255	1:1000	
ETV6	Sigma	HPA000264	1:1000	1:50
Myc-Tag	Santa Cruz	sc-40		1:50
Flag-Tag	Sigma	F3165		1:50
GPS2	GeneTex	GTX117560	1:1000	1:50
HDAC3	GeneTex	GTX83173	-	1:50
Mouse normal IgG	Santa Cruz Biotechnology	sc-2025; RRID: AB_737182	-	
II anti mouse	Santa Cruz Biotechnology	sc-51602, RRID:AB_626603	1:2000	
II anti rabbit	Santa Cruz Biotechnology	sc-2004, RRID:AB_631746	1:2000	
Donkey anti-Mouse IgG (H+L) Highly Cross-Adsorbed Secondary Antibody, Alexa Fluor 488	Thermo Fisher Scientific	A-21202; RRID: AB_141607		1:1000
Goat anti-Mouse IgG (H+L) Highly Cross-Adsorbed Secondary Antibody, Alexa Fluor 568	Thermo Fisher Scientific	A-11031; RRID: AB_144696		1:1000
Goat anti-Rabbit IgG (H+L) Cross-Adsorbed Secondary Antibody, Alexa Fluor 568	Thermo Fisher Scientific	A-11011; RRID: AB_143157		1:1000

The nuclei/cytoplasmatic localization was analyzed on ~200 cells for each condition/experiment.

## Data Availability

Data from this study are either included within the manuscript or can be obtained by contacting the corresponding author.

## Supplementary Materials

The following are available online at https://www.mdpi.com/article/10.3390/cells14010023/s1, Supplementary Figure S1. ETV6 does not control ANKRD26 mRNA expression in HEK293T. Supplementary Figure S2. ANKRD26 and ETV6 control MMP3 mRNA expression in DAMI. Supplementary Figure S3. GPS2 mediates the ANKRD26 and ETV6 interaction.
