# Supplementary material for: Inherited Thrombocytopenia Related Genes: GPS2 Mediates the Interplay Between ANKRD26 and ETV6
Source: Cells. 2024 Dec 30;14(1):23. doi: 10.3390/cells14010023 (PMC11720448; doi:10.3390/cells14010023)
Supplement: Supplementary file 1 [file cells-14-00023-s001.zip › cells-3358804-supplementary.pdf]

suppl. FIGURE 1

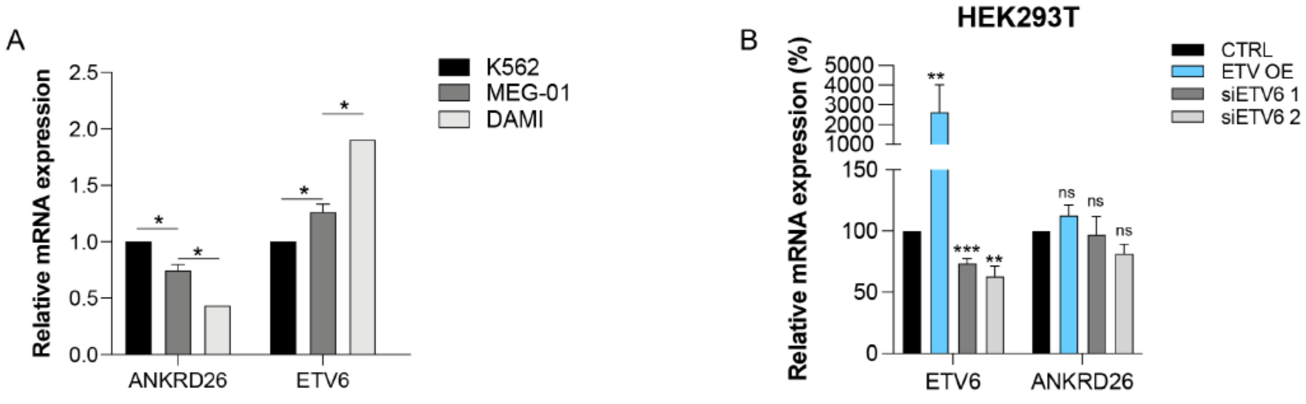

Supplementary Figure 1. ETV6 does not control ANKRD26 mRNA expression in HEK293T. **(A)** Expression levels of ANKRD26 and ETV6 determined by qRT-PCR and normalized to those of beta-Actin in K562, MEG-01 and DAMI cells. **(B)** mRNA expression level of the ANKRD26, ETV6 analyzed as in A in HEK293T cells upon overexpression of ETV6 or silencing of ETV6 using two specific siRNAs.

suppl. FIGURE 2

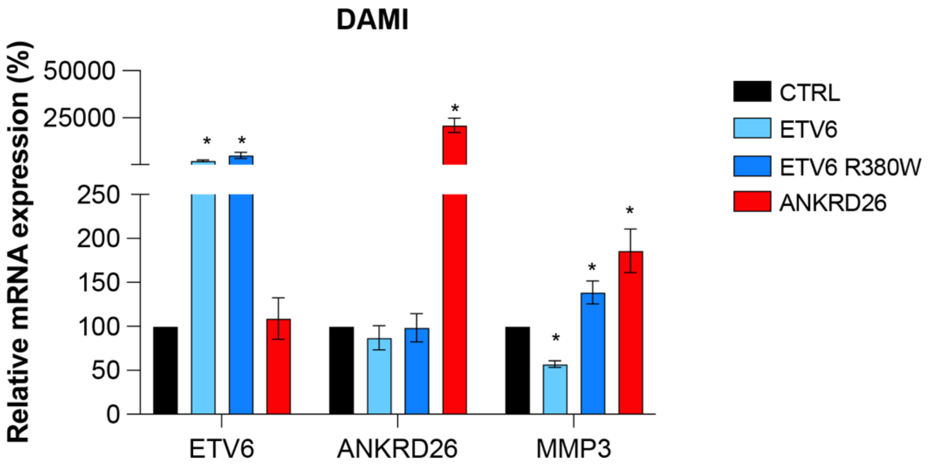

Supplementary Figure 2. ANKRD26 and ETV6 control MMP3 mRNA expression in DAMI. Expression levels of ANKRD26, ETV6 and MMP3 determined by qRT-PCR and normalized to those of beta-Actin in DAMI cells upon overexpression of overexpression of ANKRD26, and ETV6 wt or mutated as indicated. Graph represents the mean +/− SEM of three independent experiments. P value (*$p < 0.05$) was calculated by two-tailed unpaired Student's t-test.

suppl. FIGURE 3

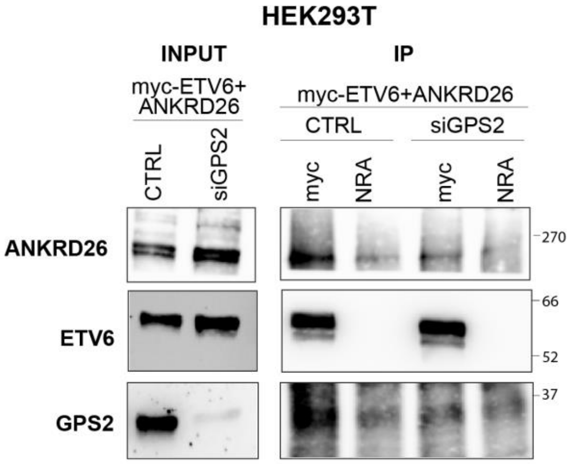

Supplementary Figure 3. GPS2 mediates the ANKRD26 and ETV6 interaction. Western blot analysis of immunoprecipitation of ETV6 with ANKRD26 and GPS2 in HEK293T cells co-expressing FLAG-ANKRD26 and myc-ETV6, as well as GPS2-silenced with a specific siRNA. NRA: not related antibody.
